# New insight into the taxonomic resolution of the genus *Bythotrephe*s Leydig (Crustacea: Cladocera) based on molecular data from Central Europe

**DOI:** 10.1038/s41598-021-02648-7

**Published:** 2021-11-30

**Authors:** Maciej Karpowicz, Magdalena Świsłocka, Joanna Moroz, Łukasz Sługocki

**Affiliations:** 1 Department of Hydrobiology, Faculty of Biology, University of Białystok, Ciołkowskiego 1J, 15-245 Białystok, Poland; 2 Department of Zoology and Genetics, Faculty of Biology, University of Białystok, Ciołkowskiego 1J, 15-245 Białystok, Poland; 3 Department of Hydrobiology, Institute of Biology, University of Szczecin, Felczaka 3C, 71-712 Szczecin, Poland

**Keywords:** Evolution, Molecular biology, Zoology, Limnology

## Abstract

The taxonomic status of the genus *Bythotrephes* Leydig (Crustacea: Cladocera) has been debated since the second half of the XIX century. The most widespread view of recent decades has been that *Bythotrephes* is a monotypic genus, which was support by preliminary molecular data. However, the recent detailed morphological revision of this genus clearly distinguishes at least seven species. Therefore, we performed a multi-lake survey in Central Europe to give new insight into the taxonomic status of *Bythotrephes* by combining genetic analysis with traditional morphology-based taxonomy. Based on the morphology we identified two species in Central Europe*, B. brevimanus* and *B. lilljeborgi*, as well as hybrid forms. For the genetic analysis, we used newly obtained 113 sequences of mtDNA COI gene of the 535-bp length *Bythotrephes* from Central Europe and sequences downloaded from GenBank. There were no significant differences between all analyzed sequences, which supports the hypothesis that *Bythotrephes* is a monotypic genus, with only one highly polymorphic species. On the other hand, the results of our work could point out that the COI gene is insufficient to evaluate the taxonomic status of *Bythotrephes*. Nonetheless, we have identified 29 new haplotypes of mtDNA COI, and one which was the same as the haplotype found in North America and Finland. Furthermore, this haplotype was the source variant from which most other haplotypes were derived.

## Introduction

The spiny waterflea genus *Bythotrephes* Leydig (Crustacea: Cladocera) is a predatory, cladoceran native to Northern Eurasia, where inhabits mostly oligo- and mesotrophic lakes. Its patchy distribution in Central Europe and in prealpine lakes is typical for ice age relicts^1,2^. In 1970s or 1980s it invaded North American lakes and is currently considered as one of the most invasive zooplankton species^3^, that has established in more than 150 lakes in Canada and the United States^4^. In Europe, *Bythotrephes* has also invaded lakes and reservoirs in the non-glaciated part of Germany, Belgium, Netherland^5,6^, but generally it is more common in Central Europe^7^.

The taxonomic status of the genus *Bythotrephes* has been debated since the second half of the XIX century^8–10^. The most widespread view of recent decades has been that *Bythotrephes* is a monotypic genus, represented by only a single morphologically highly variable species, *B. longimanus*. However, the recent detailed morphological revision of this genus clearly distinguished at least seven morphospecies spread throughout Eurasia^10,11^. The *B. longimanus* s. str. represents the relict populations in the prealpine regions^11,12^. Most *Bythotrephes* species and forms are concentrated in north and central Europe, which coincides with the region of the maximum Pleistocene glaciation^10^. In northern Europe, the ranges of *B. brevimanus*, *B. lilljeborgi*, *B. arcticus*, *B. cederströmii* at least partly overlap, and also some hybrids between these species were detected based on morphological analyses^10,13^. The most frequently found species in Central Europe are *B. brevimanus* followed by *B. lilljeborgi*^7,10^. The intensive speciation in this region might be due to the presence of numerous lakes which serve as glacial refugia during the Late Pleistocene, connections and disconnections of river basins, and variability of recolonization routes in the past^10,14,15^. Therefore, Northern Europe seems to be the primary speciation center of the genus, and it probably spread eastward from this region during the last glaciation reaching the extreme areas of North-East Asia, but it is not widely distributed there and did not penetrate North America from the west^10^. However, recent research indicated the presence of *Bythotrephes* in lakes sediments in North America dated by at least the early 1900s^16^.

The morphological traits are well described for each species, however, the genetic data are fragmentary and ambiguous. Allozyme studies from Russia indicate that the genus consists of several geographical groups, which could be separated by both morphological and genetic characters^17,18^. Based on these results, it was proposed that there were few species in the genus. This point of view was recently accepted^10,11,19^. On the other hand, allozyme studies from North America indicate a low genetic differentiation between *B. longimanus* and *B. cederströmii*^20–22^. Furthermore, Berg and Garton (1994) found that allozyme variation among sympatric populations of *B. longimanus* and *B. cederströmii* was even larger than that among allopatric populations, and they first suggested the existence of only one species, *B. longimanus*^21^. However, allozyme studies are based on protein sequences, and owing to multiple coding patterns, species-based variation in DNA sequences might not be detected^1^. Therriault et al. (2002) used sequencing of the mitochondrial COI gene to explore the genetic nature of these species. They analyzed *B. cederströmii* (2 lakes in Canada), *B.* cf. *brevimanus* (1 lake in Finland, 2 lakes in Germany) and *B. cederströmii connectens* from the Volga River in Russia, which seems to be a hybrid form. These results revealed low genetic differentiation between these species and they suggest that *Bythotrephes* is a monotypic genus with a single species, *Bythotrephes longimanus* Leydig, 1860. Furthermore, the same haplotype was identified in Lake Ontario and Lake Puruvesi (Finland) suggesting a potential source of invasion from the Baltic Sea region^1^.

Nevertheless, the records of *Bythotrephes* are scattered and incomplete, especially in Europe where most species or forms occur. Therefore, we performed morphological and genetic analyses of *Bythotrephes* populations in 20 lakes in Central Europe (NW and NE Poland, Lithuania). Our study aims to present the intra- and interpopulation genetic variability of *Bythotrephes* in Central Europe. We also present morphological differences between *B. brevimanus* and *B. lilljeborgi*. Furthermore, photography documentation of each individual together with its COI sequence was uploaded to the BOLD System. We believed that our molecular data combined with available sequences will allow a better understanding of phylogeographic patterns of this genus and may provide new insight into its history of invasion in North America.

## Results

### Morphology analysis

**Figure 1:**
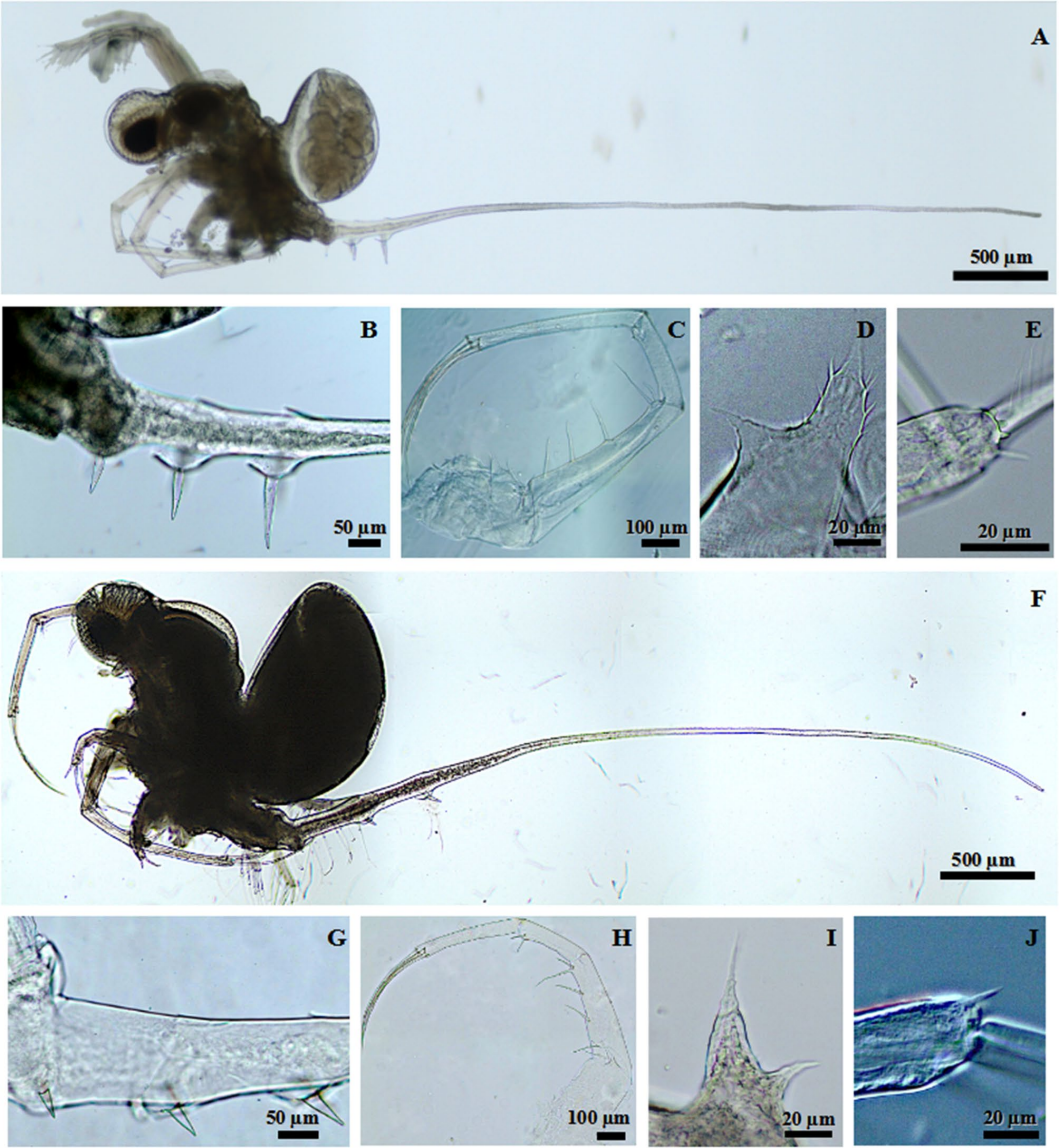
*Bythotrephes lilljeborgi* from Lake Białe Wigierskie (**A**–**E**) and *B. brevimanus* from Lake Drawsko (**F**–**J**) with morpho-functional traits distinguishing species [body, general lateral view—(**A**, **F**); claws of postabdomen and caudal process—(**B**, **G**); thoracic limbs of first pair with their armament—(**C**, **H**); pseudognathobasic process on the protopodite of thoracic limbs of first pair—(**D**, **I**); apical end of upper antennal branches—(**E**, **J**)].

Based on the morphological features we identified in Central Europe two species *B. brevimanus* and *B. lilljeborgi* (Fig. 1) following keys of Korovchinsky^10,11,23^, and possible hybrid between these species. *B. lilljeborgi* was found in three lakes in NE Poland (Gaładuś, Białe Wigierskie and Hańcza) and one in Lithuania (Galstas). *B. brevimanus* was found in three lakes of NW Poland (Drawsko, Żerdno, Lubie) and in Lake Studzieniczne (NE Poland). The possible hybrids between these species were found in other lakes, while in eastern part were more like *B. lilljeborgi* and in west Poland were more like *B. brevimanus*.

The morphological differences between both species included few morphological traits, like claws of postabdomen and caudal process, thoracic limbs of first pair (tl I) with their armament, and apical end of antennal branches. The claws of postabdomen and caudal process in *B. lilljeborgi* are comparatively long (4.8–11.6% of body length) and directed downwards, while in *B. brevimanus* they are short (2.0–6.9% of body length) and directed backward (Fig. 1B, G). The *B. brevimanus* have generally shorter tl I with a shorter distal segment (Fig. 1H), while in *B. lilljeborgi* the tl I and distal segment are longer (Fig. 1C). The apical setea of the second segment of tl I in *B. lilljeborgi* are reduced, while in the *B. brevimanus* are better developed (Fig. 1C, H). On the protopodite of tl I, pseudognathobasic process armed lateraly and distaly with two outgrowths with apical setae in both species, but in *B. lilljeborgi* distal outgrowth with numerous spinules (Fig. 1D, I). The distal segment of upper antennal branches with small apical spine in both species, but in *B. lilljeborgi* also with denticles (Fig. 1F, J). The hybrid was characterized with varied morphological traits for both species. Some of them have smaller claws of postabdomen directed downward and backward (like *B. brevimanus*), but the tl I and swimming antennal like *B. lilljeborgi*. Others have larger claws of postabdomen directed downward, but tl I was similar to *B. brevimanus*.

However, morphological data was inconsistent with genetic (COI) data. We observed different morphological traits for populations with the same haplotype (H7). Population from Lake Białe Wigierskie (NE Poland) had morphological traits similar to *B. lilljeborgi* like comparatively long and massive claws of postabdomen and caudal process inserted densely (Fig. S1B), comparatively longer tl I and distal segment of tl I (Fig. S1C), and smaller body size (Fig. S1A). While the same haplotype (H7) from Lake Słowa (NW Poland) had morphological traits similar to *B. brevimanus* like smaller claws of postabdomen and caudal process inserted comparatively sparsely (Fig. S1F), shorter tl I and distal segment of tl I (Fig. S1E), and larger body size (Fig. S1D).

### Genetic analysis

**Figure 2:**
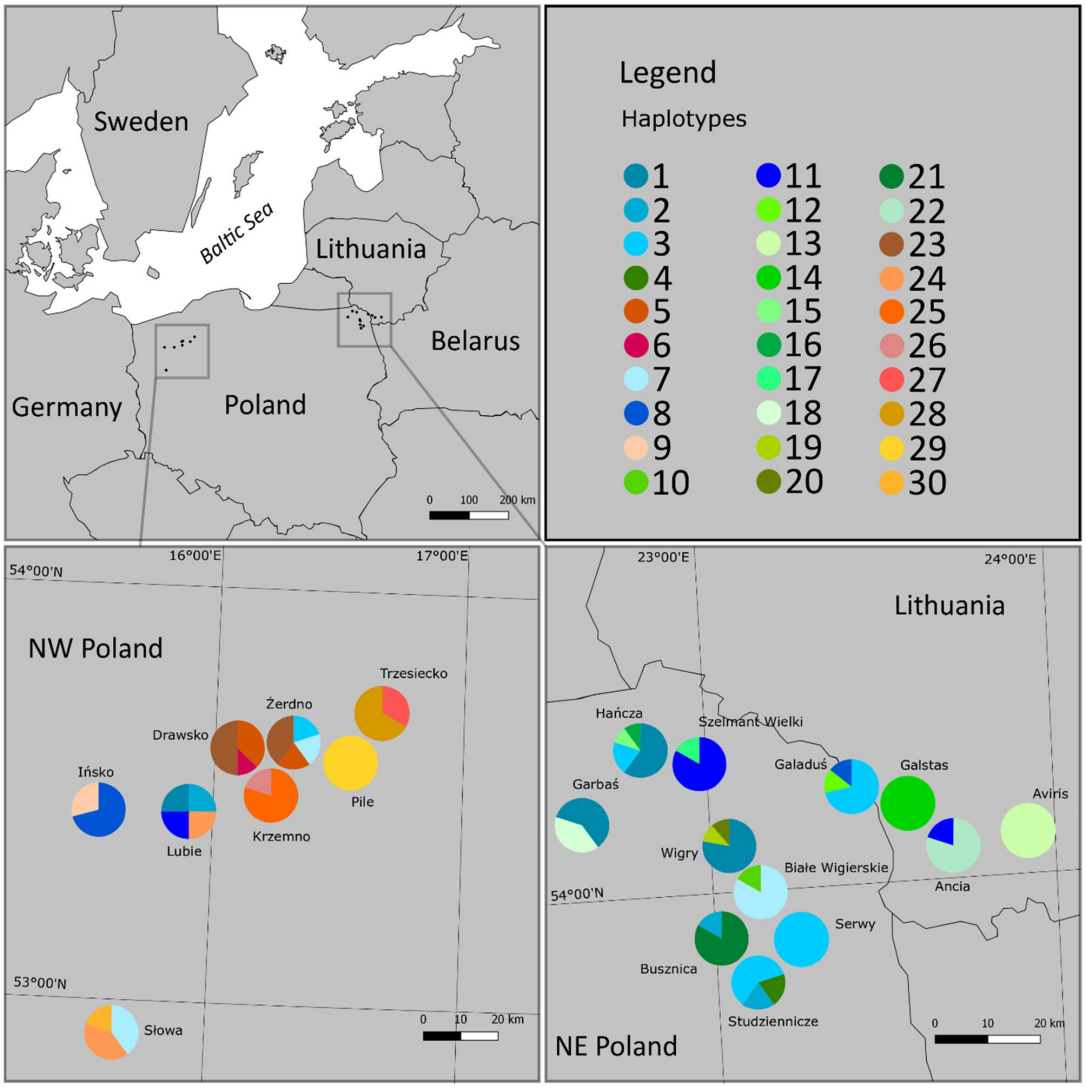
Map of the studied lakes in Central Europe with *Bythotrephes* haplotypes distribution and frequencies.

Our analysis of a 535-bp mitochondrial DNA cytochrome c oxidase subunit 1 (COI) gene fragment amplified from 113 *Bythotrephes* individuals from 17 studied lakes in Poland and 3 lakes in Lithuania yielded 45 polymorphic sites with a total of 37 transitions and 8 transversions, defining 30 mtDNA COI haplotypes (Fig. 2; GenBank accession numbers for obtained haplotypes: MZ196458–MZ196487). Twenty-nine mtDNA COI gene haplotypes were detected for the first time, while only one haplotype from our study (H8) was identical as the haplotype previously described by Therriault et al. (2002; GenBank acc. num. AF435122 described as *B*. *longimanus*, probably *B. cederströmii*), deWaard et al. (2006; GenBank acc. num. DQ310655 for *B. cederströmii*)^1,24^, and two unpublished sequences described as *B*. *longimanus* (GenBank acc. num. GU689226 and HQ966461). In total, the 20 studied lakes haplotypes and nucleotide diversity were very high (*h* = 0.94, *π* = 1.30%; Table 1) and the number of mtDNA COI haplotypes ranged from 1 (Lake Serwy in NW Poland, and lakes Galstas and Aviris in Lithuania) to 4 (lakes Lubie and Żerdno in NW Poland, and Lake Hańcza in NE Poland) (Fig. 2; Table 1). The highest genetic diversity was found in the lakes Lubie and Żerdno in NW Poland (*h* = 1.00, 0.90; *π* = 1.59, 1.05, respectively), whereas the corresponding values (*h* and *π* = 0) were the lowest in lakes Serwy (NE Poland), Galstas and Aviris (Lithuania). Although the number of haplotypes per lake did not exceed five, the number of segregating sites within studied lakes ranged from 0 to 15 (Lake Lubie). Values of the mean number of pairwise differences differed from 0 to 8.50 (Lake Lubie; Table 1).

**Table 1:** Mitochondrial DNA cytochrome c oxidase subunit 1 (COI) gene (535-bp) diversity indices for 20 *Bythotrephes* lakes in Poland and Lithuania.

No	Lake	*N*	*Nh*	*h* (SE)	*π* (SE)	*S*	PD
**North-West Poland**							
1	Słowa	5	3	0.80 (± 0.16)	0.30 (± 0.25)	3	1.60 (± 1.13)
2	Ińsko	7	2	0.48 (± 0.17)	0.09 (± 0.09)	1	0.48 (± 0.46)
3	Lubie	4	4	1.00 (± 0.18)	1.59 (± 1.11)	15	8.50 (± 4.99)
4	Krzemno	5	2	0.40 (± 0.24)	0.75 (± 0.52)	10	4.00 (± 2.40)
5	Drawsko	8	3	0.68 (± 0.12)	0.15 (± 0.13)	2	0.79 (± 0.63)
6	Żerdno	5	4	0.90 (± 0.16)	1.05 (± 0.71)	13	5.60 (± 3.23)
7	Pile	1	1*	1.00 (± 0.00)	0.00 (± 0.00)	0	0.00 (± 0.00)
8	Trzesiecko	3	2	0.67 (± 0.31)	0.50 (± 0.45)	4	2.67 (± 1.92)
**North-East Poland**							
9	Szelment Wielki	6	2	0.33 (± 0.22)	0.06 (± 0.08)	1	0.33 (± 0.38)
10	Garbaś	5	2	0.60 (± 0.17)	0.34 (± 0.27)	3	1.80 (± 1.24)
11	Hańcza	10	4	0.64 (± 0.15)	0.75 (± 0.46)	11	4.00 (± 2.18)
12	Białe Wigierskie	6	2	0.33 (± 0.22)	0.06 (± 0.08)	1	0.33 (± 0.48)
13	Busznica	6	2	0.33 (± 0.21)	0.19 (± 0.17)	3	1.00 (± 0.77)
14	Wigry	9	3	0.42 (± 0.19)	0.45 (± 0.30)	10	2.39 (± 1.43)
15	Studzienniczne	5	3	0.70 (± 0.22)	0.15 (± 0.15)	2	0.80 (± 0.68)
16	Serwy	3	1	0.00 (± 0.00)	0.00 (± 0.00)	0	0.00 (± 0.00)
17	Gaładuś	7	3	0.52 (± 0.21)	0.87 (± 0.56)	11	4.67 (± 2.60)
**Lithuania**							
18	Galstas	7	1	0.00 (± 0.00)	0.00 (± 0.00)	0	0.00 (± 0.00)
19	Ancia	5	2	0.40 (± 0.24)	0.67 (± 0.48)	9	3.60 (± 2.19)
20	Aviris	6	1	0.00 (± 0.00)	0.00 (± 0.00)	0	0.00 (± 0.00)
	Total	113	30	0.94 (± 0.01)	1.30 (± 0.68)	45	6.95 (± 3.29)

N—sample size; Nh—number of haplotypes; h—haplotype diversity; π—nucleotide diversity (%); S—number of segregating sites; PD—mean number of pairwise differences; SE—standard error; *—due to only one individual, Pile Lake was not included in statistical comparisons.

**Figure 3:**
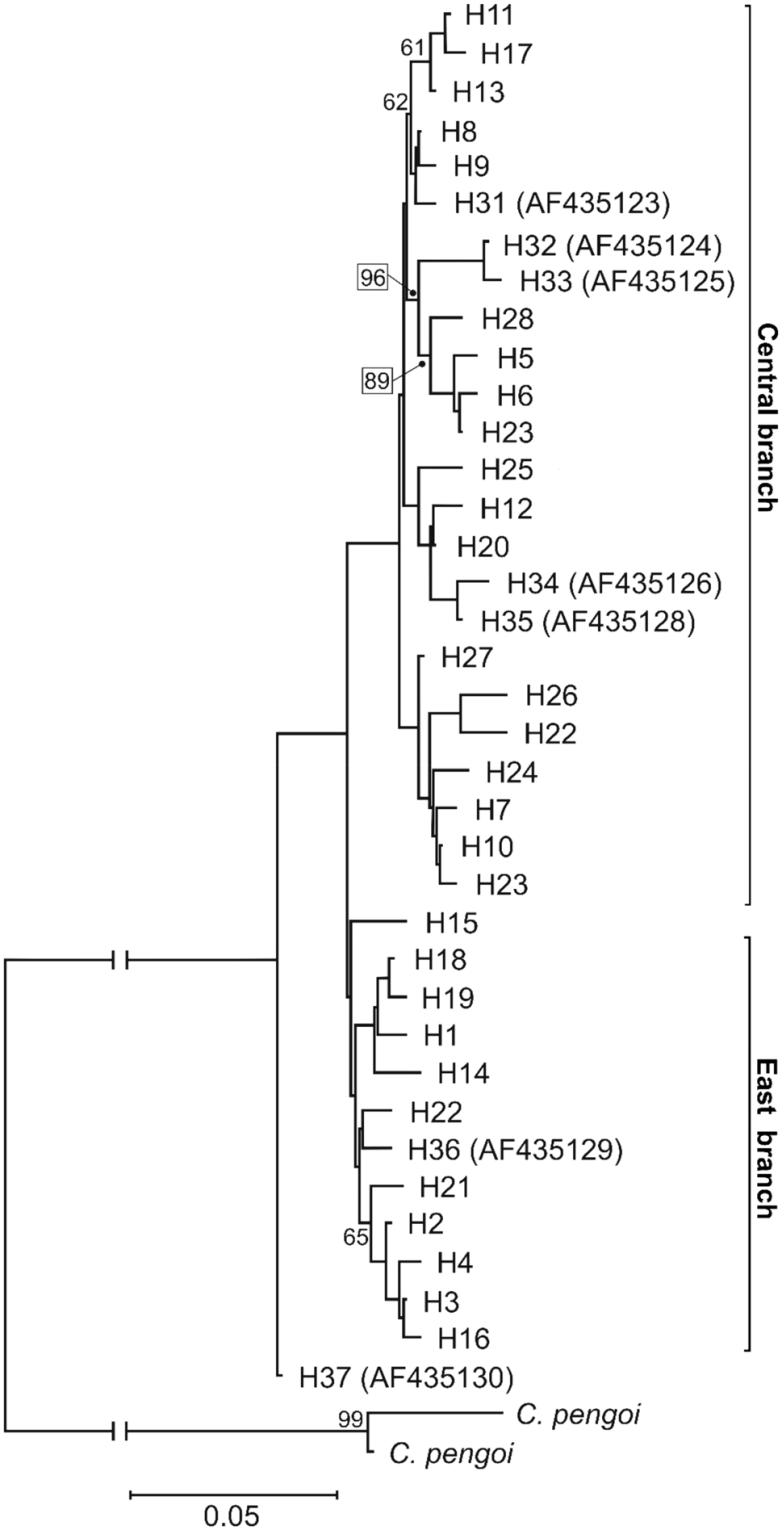
Maximum-likelihood tree computed with the GTR + I + G model of sequence evolution, representing phylogenetic relationships among the mtDNA COI sequences found in studied *Bythotrephes* lakes from Poland, Lithuania and downloaded from GenBank (Therriault et al., 2002). Numbers listed at nodes represent percent support for that node from 1000 bootstrap replicates. The tree has been rooted with sequences of *Cercopagis pengoi*.

**Figure 4:**
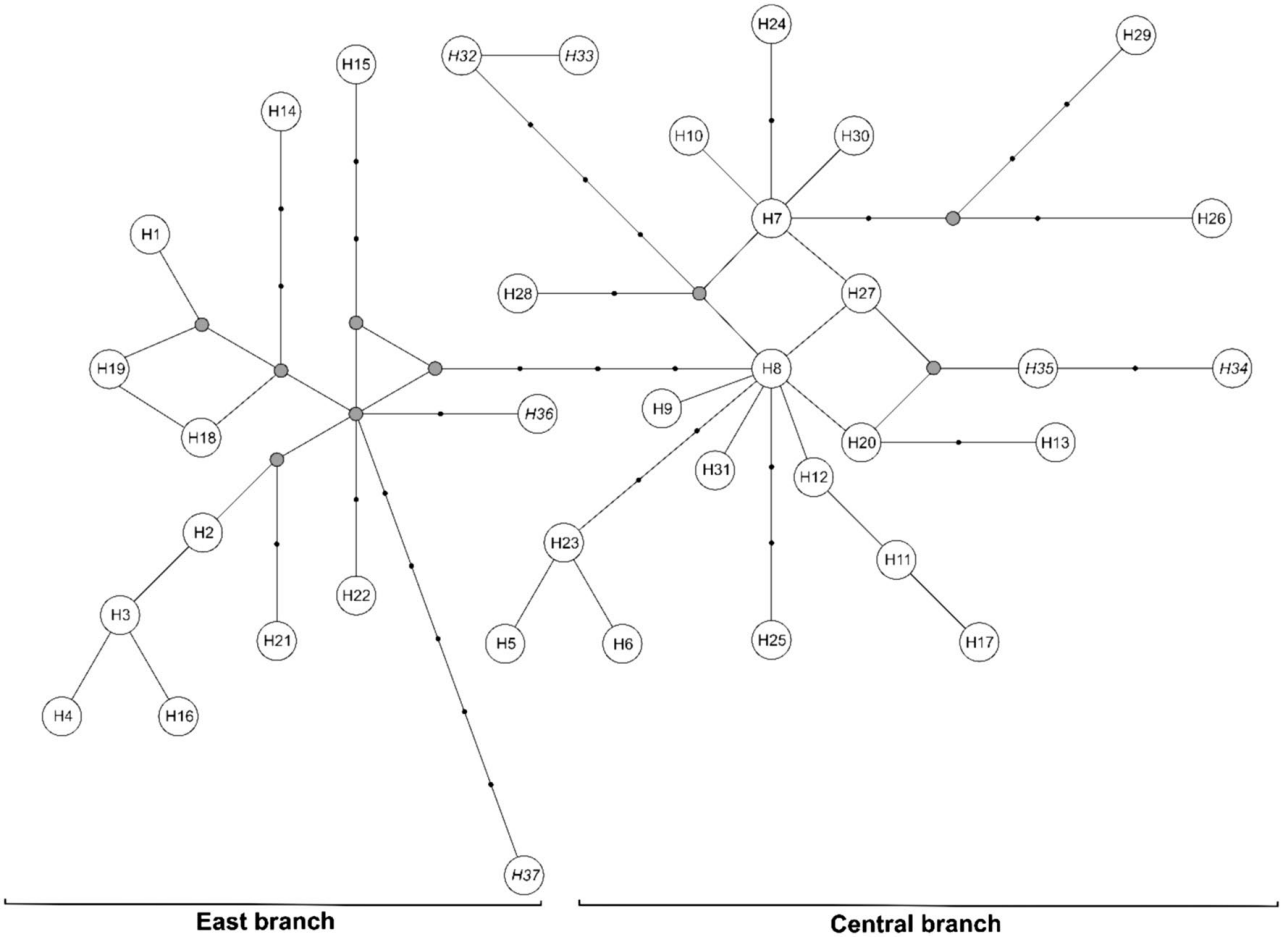
Median-joining network of mtDNA COI haplotypes of the genus *Bythotrephes* belonging to the East and Central branch. Thirty haplotypes found in this study have letter codes with the number from H1 to H30, while haplotypes downloaded from GenBank are in italics *H31*–*H37* (*H31*—Finland; *H32*, *H33*, *H34*—Germany; *H35*—Netherlands; *H36*, *H37*—Volgograd Reservoir, Russia). Missing haplotypes are indicated by a grey dot.

The mtDNA COI haplotypes of genus *Bythotrephes* were used to infer the phylogenetic tree. The ML phylogenetic analyses revealed the presence of the two evolutionary branches: East and Central (Fig. 3), and the net divergence (*d*_A_) between branches was 0.7% (SE ± 0.3%). The majority of haplotypes found in NE Poland created the East branch, together with haplotypes H14 and H22 found in the Lithuania lakes (Figs. 3 and 4), which seems to be *B. lilljeborgi*, or hybrid *B. lilljeborgi* × *B. brevimanus*. Only haplotypes H1, H2, and H3 from this branch were found in two lakes from NW Poland (Lubie and Żerdno). Haplotype H36 (AF435129) recorded in the Volgograd Reservoir by Therriault et al. (2002) was grouped also in the East branch and was described as *B. cederströmii connectens*^1^. The Central branch grouped *Bythotrephes* haplotypes found in all lakes from NW Poland, lakes Ancia (H11) and Aviris (H13) in Lithuania, and 7 haplotypes recognized in NE Poland lakes (H7 and H10 from Białe Wigierskie; H8, H12, and H17 from Gaładuś; H11—Szelment Wielki; H20—Wigry) (Figs. 3 and 4). The Central branch seems to represent *B. brevimanus* and also hybrid *B. lilljeborgi* × *B. brevimanus*. In this phylogenetic branch, the position of the H8 haplotype indicates that it was the sequence variant source from which a significant number of haplotypes were derived (Fig. 4). Interestingly, this haplotype, apart from the Polish lakes Ińsko (NW Poland) and Gaładuś (NE Poland), was previously described in Canada Lake Simcoe and Ontario, and in Finland Lake Puruvesi (Therriault et al. 2002; AF435122) and it was identified as *B. cederströmii*. This phylogenetic branch also grouped haplotypes described by Therriault et al. (2002) in the European part of *Bythotrephes* range: Finland (H31—AF435123), Germany (H32—AF435124, H33—AF435125, H34—AF435126), Netherlands (H35—AF435128) which seems to be *B. brevimanus*. Therefore, our Central branch groups with other lakes in Central Europe with the domination of *B. brevimanus*, as well as with *B. cederströmii* from Canada and Finland. The East branch seems to be *B. lilljeborgi* and is grouped also with Volga River which was described as *B. cederströmii connectens* (Fig. 4). However, the photography provided by Therriault et al. (2002) may rather suggest hybrid *B. lilljeborgi* × *B. cederströmii* in Volga River^1^.

The haplotype network based on the mtDNA COI sequences revealed the presence of the same *Bythotrephes* phylogenetic branches (Fig. 4). At least 6 mutations distinguished haplotype H8 from the Central branch from haplotypes (H22 and H36) belonging to the East branch. The East branch seems to group *B. lilljeborgi* from NE Poland and Lithuania, with Volga River (Fig. 4). However, the haplotype 37 from the Volga River is distinguished by at least 8 mutations (Fig. 4).

The most common mtDNA COI haplotypes H1 and H3 occurred with a frequency of 15% and 12.4%, respectively. The H1 haplotypes was found in Lake Lubie in NW Poland and lakes Garbaś, Hańcza, Wigry in NE Poland. In Lake Żerdno from NW Poland and lakes Hańcza, Studzienniczne, Serwy, Gaładuś in NE Poland the haplotype H3 was detected. Thirteen haplotypes (5 from NW Poland and 8 from NE Poland) were found only in one individual (Table S1). While haplotypes H13 and H14 occurred in all analyzed *Bythotrephes* taken from lakes Aviris and Galstas in Lithuania. Haplotypes 9, 18, 21, 22, 25 and 28 were found in 2–5 individuals, but each of them occurred in only one lake (Ińsko, Garbaś, Busznica, Ancia, Krzemno, and Trzesiecko; respectively) (Fig. 2; Table S1).

**Figure 5:**
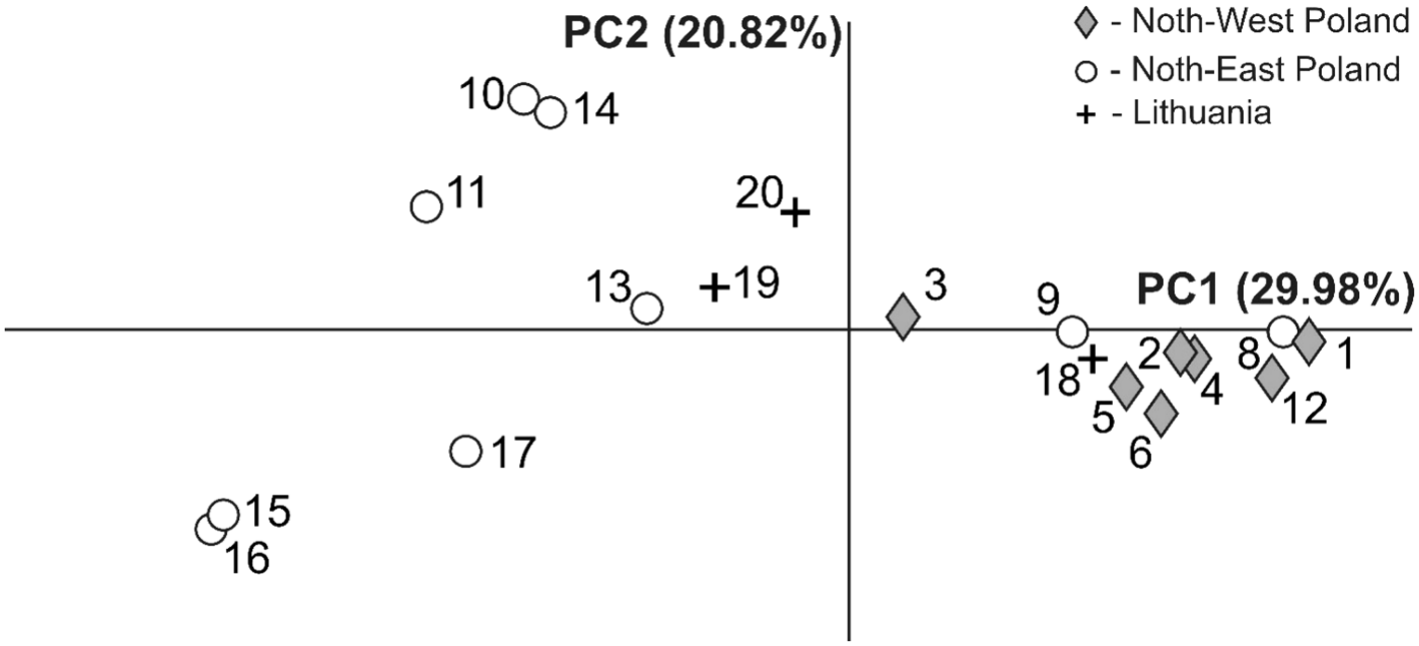
Principal coordinate analysis performed on pairwise *Φ*_ST_ values of the studied *Bythotrephes* lakes in Poland and Lithuania. The numbers of lakes 1–20 following the Table 1.

Pairwise genetic differentiation values (mtDNA COI) between studied lakes ranged from 0 to 1.00 (Serwy–Galstas, Serwy–Aviris, Galstas–Aviris). The majority of comparison values of *Φ*_ST_ were significant (Table S2). The first and second axes of the PCA (PC1 and PC2) performed on the data set containing 19 lakes (Lake Pile was excluded from the analysis due to being represented by only one individual) explained 29.98 and 20.82% of the total variability (Fig. 5). The analysis based on *Φ*_ST_-matrix separated almost all NE Poland lakes (except Szelment Wielki and Białe Wigierskie) from lakes localized in NW Poland and Lake Galstas in Lithuania. Studied lakes were in general separated into two groups along PC1 according to their possessing mtDNA COI representing the Central and East phylogenetic branches. The intermediate form was found in lake Lubie, where haplotypes belonging to both ML tree branches were described. With respect to the PC2 the most divergent were NE Poland lakes Garbaś, Wigry, Studzienniczne and Serwy (Fig. 5).

## Discussion

The taxonomic status of the genus *Bythotrephes* has been debated for a long time and now is intensified due to contrary data from genetic and morphological analyses. The morphology data generally indicated the presence of few species within the *Bythotrephes* genus. By combining molecular data (COI gene) with the morphological description we want to give new insight into the taxonomic status of *Bythotrephes*. At first in the second half of the XIX century, two species *B. longimanus* and *B. cederströmii* were described, and this point of view is the most widespread and included in the taxonomic keys to freshwater zooplankton^25,26^. Intensive morphological investigation of the *Bythotrephes* genus together with allozyme analysis by Litvinchuk and Litvinchuk (2016) revealed the presence of five species: *B. longimanus*, *B. cederströmii*, *B. arcticus* (“*B. crassicaudus*”), *B. brevimanus*, and *B. transcaucasicus*^18^. This revision was continued by Korovchinsky (2015, 2018) which described new species of the genus, *B. lilljeborgi* and *B. centralasiaticus*, as well as performed redescriptions of several species^10,11,23^. As a result, few morphological traits were described which differ between these species. However, the morphological data also indicated the common presence of the interspecific hybrid forms: *B. brevimanus* × *B. cederströmii*; as well as rare forms *B. arcticus* × *B. cederströmii*, and *B. centralasiaticus* × *B. cederströmii* found in single localities^27^. We describe the new hybrid form *B. lilljeborgi* × *B. brevimanus*, which were common in the study area, where the range of both species overlaps. These hybrids were characterized by varied morphological traits for both species.

The first suggestion for considering *Bythotrephes* as a monotypic genus comes from the second half of the XX century from Rybinsk Reservoir of the Volga River where highly variable populations of either *B. longimanus* or both *B. longimanus* and *B. cederströmii* were found and possessing intermediate features. This makes it impossible to distinguish species based on morphology and leads to a conclusion of the existence of only one highly variable species, *B. longimanus*^9,28–30^. Similarly to our study where hybrid forms were very common. Furthermore, we observed different morphological traits for populations with the same haplotype. The allozyme studies from North America also indicate low genetic variability between *B. longimanus* and *B. cederströmii*^20–22^ contrary to Litvinchuk and Litvinchuk (2016)^18^. However, according to Korovchinsky and Arnott (2019) only one species *(B. cederströmii*) is present in North America^13^. Nevertheless, Berg and Garton (1994) found that allozyme variation among sympatric populations of *B. longimanus* and *B. cederströmii* was larger than that among allopatric populations, and they also suggested the existence of only one species (*B. longimanus*)^21^. We also have found large interpopulation genetic diversity (COI), and in one lake even four haplotypes were found. The first results of sequencing COI gene by Therriault et al. (2002) indicated a large genetic similarity of *Bythotrephes* forms in North American and Europe and give better proof for *Bythotrephes* as a monotypic genus^1^. However, their study includes only a few sequences from North America and Europe. Therefore, we performed a multi-lake survey in Central Europe to give new insight into the taxonomic status of *Bythotrephes* by combining genetic analysis with traditional taxonomy. We identified two species in Central Europe, *B. brevimanus* and *B. lilljeborgi*, and also a hybrid between these species. For phylogenetic studies, we used our 113 sequences from Central Europe (Poland, Lithuania) with sequences from Finland, Germany, Netherland (seems to be *B. brevimanus*), and Canada (seems to be *B. cederströmii*). There were no significant differences between all analyzed sequences of COI gene, even though we analyzed three species (*B. brevimanus*, *B. lilljeborgi*, *B. cederströmii*) identified based on the morphology. Therefore, our data also support the hypothesis on the monotypic genus of *Bythotrephes*, with only one highly polymorphic species. The fragment of the mitochondrial COI gene was found to be an informative molecular marker for such studies, and COI data were successfully used for taxon delimitation in cladocerans^31,32^. At the same time, mitochondrial uniformity could be a consequence of hybridization and old introgression. Moreover, COI is a tricky gene—sometimes it has no resolution to discriminate two apparent species (Kotov, 2021, pers. comm., 8 May). The results of our work pointed out that the COI gene is insufficient to evaluate the taxonomic status of *Bythotrephes*. Most morphological characters are related to the activity of nuclear genes, therefore further analysis of nuclear genes could be more informative for the *Bythotrephes* genus. Nevertheless, according to morphology, one species seems quite improbable, because morphological differences, at least between some species, are very conspicuous (Korovchinsky 2021, pers. comm., 8 May).

The level of gene flow among populations may be a function of dispersal ability^33^. Research suggests that widely dispersing species have high intrapopulation variation and low interpopulation variation^34,35^, while poorly dispersing species show the opposite pattern^33^. Therefore, large intrapopulation variability in our study indicated the wide dispersion abilities of *Bythotrephes*. Nowadays this species is considered one of the most invasive zooplankton member since they invaded North American^3,4^. *Bythotrephes* was first observed in North America in Lake Ontario in 1982^36^ and was probably introduced via ballast water in ships that travel between St. Petersburg in Russia, and Great Lakes ports^37^. A study of Berg et al. (2002) based on allozymes suggested Lake Ladoga in Russia as the source of the invasion, thereby first studies have identified only a single source region somewhere in the northeast Baltic region^22^. Therriault et al. (2002) identified the same haplotype of the mtDNA COI gene in Lake Puruvesi in Finland and indicated that this region is likely the source of the North American invasion^1^. We have identified the same haplotype in two Polish lakes, Ińsko (NW Poland) and Gaładuś (NE Poland). Furthermore, this haplotype (H8) in our median-joining network (Fig. 3) was the source variant from which a significant number of haplotypes were derived. The other haplotypes in Central Europe have high similarity to H8, while north-eastern Poland and Lithuania lakes were more similar with Volga River and formed East Branch. Therefore, our results pointed that central Baltic countries (Germany, Poland) could be a more accurate source of the invasion, than the northeast Baltic region. This pattern of gene flow corresponding with major sea routes connecting the Great Lakes with the North Sea and Baltic Sea countries^38^.

## Methods

### Field work

We collected the *Bythotrephes* samples in the middle of summer (2018 and 2020) from 20 lakes in Central Europe (NW Poland—8, NE Poland—9, Lithuania—3; Fig. 2). The samples from each lake were collected in triplicate by vertical hauls with a 100-µm plankton net close to the deepest point of lakes or at a location with a depth exceeding 15 m in large and deep lakes. Concentrated samples were added into a 110-ml tube and fixed in 96% ethanol. Individuals of *Bythotrephes* were separated from other plankton using light microscopy. Photographic documentation was made for each individual for genetic analysis and it is available at BOLDSystem (project code: BYTHO; BIN: BOLD:AAB9254; sample IDs: UwB_1_Bl1, UwB_2_Bl2, …, UwB_113_Bl113). *Bythotrephes* individuals for genetic analysis were selected and stored in 70% ethanol at 4 °C before DNA extraction. The map of the studied lakes in Central Europe with *Bythotrephe*s haplotypes distribution and frequencies was created in QGIS software (version 2.18.24; www.qgis.org).

### Morphological analysis

The species identification was based on the recent review of this genus by Korovchinsky^10,11,18,23^. We present the most important morpho-functional traits which differ between identified species, like claws of postabdomen, thoracic limbs of first pair (tl I) with their ornamentation^10^, pseudognathobasic process on the protopodite of tl I, and apical end of upper antennal branches. Additionally, we performed detailed measurements of body and body parts measurements according to the protocol proposed by Korovichinsky^11,23^ for two populations from Lake Drawsko and Galstas. Lake Galstas represent *B.* cf. *lilljeborgi* and ‘East branch’ of our molecular analysis with a single haplotype (H14), which was found only in this lake (Table S1). While Lake Drawsko represents *B.* cf. *brevimanus* and ‘Central branch’ of our molecular analysis with haplotypes H5, H6, and H23. We also compared morphological differences in one haplotype (H7) from Lake Białe Wigierskie (NE Poland) and Lake Słowa (NW Poland). The morphological analysis was performed using an Olympus BX53 microscope with cellSens imaging software.

### Molecular analyses

We analyzed 113 individuals of *Bythotrephe*s from 20 different lakes in Central Europe (Fig. 2). The sample sizes from given lakes ranged from 1 (Lake Pile) to 10 individuals (Lake Hańcza; Table 1). The DNA extraction was performed with the DNeasy Blood and Tissue Kit (Qiagen, Hilden, Germany) according to the manufacturer’s instructions. PCR amplification of mitochondrial DNA cytochrome c oxidase subunit 1 (COI) gene was carried out with a GeneAmp PCR System 9700 in 5 μL volumes, and the reaction mixtures consisted of 2 μL of extracted genomic DNA as a template, 1.7 μL of Qiagen Multiplex PCR Master Mix (1x), 0.3 μL mix of newly designed primers (BytCOI_F: 5′-TCGGAATTTGAGCTGGGATAGTA-3′; BytCOI_R: 5′-TGTAAGGAGTATAGTAATAGCTCC-3′) and 1 μL of Qiagen nuclease-free water. The primers used in this study were designed in FastPCR software^39^. The reaction conditions were as follows: 15 min at 95 °C of an initial denaturation, 35 cycles with denaturation at 94 °C for 30 s, annealing at 57 °C for 90 s, extension at 72 °C for 60 s, and final elongation for 30 min at 60 C. Amplified PCR products were purified with shrimp alkaline phosphatase (SAP) and Exonuclease I (Thermo Scientific) in an enzymatic reaction following the manufacturer’s protocols. They were then processed for cycle sequencing PCR with a BigDye™ Terminator v3.1 Cycle Sequencing Kit v.3.1 (Applied Biosystems, Foster City, CA, USA) using primer forward (BytCOI_F). Unincorporated dideoxynucleotides were eliminated from the sequencing reaction using the ExTerminator Kit (A&A Biotechnology). We carried out the detection of sequencing reaction products on an ABI PRISM 3100 Genetic Analyzer (Applied Biosystems). Sequencing results were edited and aligned manually using BioEdit^40^.

### Statistical analyses

We used DnaSP v.5.10^41^ to calculate the measures of sequence variation, including the number (*Nh*) and frequency of haplotypes, haplotype diversity (*h*), nucleotide diversity (*π*, in %), the number of segregating sites (*S*), and the mean number of pairwise differences (PD). The pairwise *Φ*_ST_ values for the 19 lakes were calculated in Arlequin v.3.5.1.2.^42^, and their significance was tested using 10,000 permutations that were corrected for multiple tests by Bonferroni correction. Principal coordinate analysis (PCA) was performed on mtDNA COI *Φ*_ST_ data in Genalex v.6.0^43^. To test the phylogenetic relationships among obtained in the study mtDNA cytochrome oxidase subunit 1 haplotypes, we constructed phylogenetic trees with Mega v.6.06^44^ with 1,000 bootstrap replicates used to assess support for tree nodes. The optimal model of substitution^45^ for our mtDNA COI sequences was determined using Akaike’s information criterion^46^ with jModelTest^47^ and used to calculate a maximum-likelihood (ML) algorithm. The GTR + I + G model was selected as the best-fitting model for the ML tree, which was additionally rooted using mtDNA COI haplotypes from *Cercopagis pengoi* (AF320013 from Black Sea and AF320014 from Caspian Sea) downloaded from GenBank. We estimated net pairwise divergence (*d*_A_) between mtDNA COI branches Central and East using the program Mega v.6.06^44^. The standard error of estimates was calculated with 1000 bootstrap pseudo-replicates. The median-joining method available in Network v.10.2.^48^ was used to visualize the networks between *Bythotrephes* mtDNA COI haplotypes. To construct a median-joining network and the ML tree, apart from the mtDNA COI haplotypes obtained in our study, we used *Bythotrephes* haplotypes previously described by Therriault et al. (2002)^1^, with the following GenBank accession numbers: AF435122–AF435131. The sequence from Canada (AF 435122) seems to be *B. cederströmii* based on the photos from BOLD System (record NJCGS013-09). While the same haplotype (AF 435122) from Lake Puruvesi in Finland seems to be *B. brevimanus* based on the photography in the paper by Therriault et al. (2002)^1^. The haplotypes from Germany and Netherland (AF 435124—Post See, AF 435125—Post See, AF 435126—Selenter See, AF 435127—Selenter See, AF 435128—Petrusplaat/Dordrecht)—seems to be *B. brevimanus* based on the geographical distribution of this genus.

## Supplementary Information

Supplementary Information 1.

## References

[1] Therriault TW (2002). Taxonomic resolution of the genus *Bythotrephes* Leydig using molecular markers and re-evaluation of its global distribution. Divers. Distrib..

[2] Karpowicz M, Ejsmont-Karabin J, Kozłowska J, Feniova I, Dzialowski AR (2020). Zooplankton community responses to oxygen stress. Water.

[3] Dexter E, Bollens SM (2020). Zooplankton invasions in the early 21st century: A global survey of recent studies and recommendations for future research. Hydrobiologia.

[4] Kerfoot WC (2016). A plague of waterfleas (*Bythotrephes*): Impacts on microcrustacean community structure, seasonal biomass, and secondary production in a large inland-lake complex. Biol. Invasions.

[5] Ketelaars HAM, van Breemen LWCA (1993). The invasion of the predatory cladoceran *Bythotrephes longimanus* Leidig and its influence on the plankton communities in the Biesbosch reservoirs. Verh. Int. Ver. Limnol..

[6] Ketelaars HAM, Gille L (1994). Range extension of the predatory cladoceran *Bythotrephes longimanus* Leidig 1860 (Crustacea, Onychopoda) in western Europe. Neth. J. Aquat. Ecol..

[7] Karpowicz M, Ejsmont-Karabin J (2021). Diversity and structure of pelagic zooplankton (Crustacea, Rotifera) in NE Poland. Water.

[8] Mordukhai-Boltovskaya ED (1959). On the systematics of the genus *Bythotrephes* Leydig (Cladocera*)*. Byulleten instituta Biologii vodokhranilishch akademii nauk SSSR.

[9] Rivier IK, Dumont HJ (1998). The predatory Cladocera (Onychopoda: Podonidae, Polyphemidae, Cercopagidae) and Leptodorida of the world. Guides to the Identification of the Microinvertebrates of the Continental Waters of the World 13.

[10] Korovchinsky NM (2020). Description of a new species in the genus *Bythotrephes* Leydig, 1860 (Crustacea: Cladocera: Onychopoda), supplements to selected species, and concluding remarks on the genus. Zootaxa.

[11] Korovchinsky NM (2015). Redescription of Bythotrephes longimanus Leydig, 1860 and *B. cederströmii* Schödler, 1877 (Crustacea: Cladocera: Onychopoda), with notes on the morphology and systematics of the genus Bythotrephes Leydig, 1860. Zootaxa.

[12] Horváth Z (2017). Zooplankton communities and *Bythotrephes longimanus* in lakes of the montane region of the northern Alps. Inland Waters.

[13] Korovchinsky NM, Arnott SE (2019). Taxonomic resolution of the North American invasive species of the genus *Bythotrephes* Leydig, 1860 (Crustacea: Cladocera: Cercopagididae). Zootaxa.

[14] Hewitt G (2000). The genetic legacy of the quaternary ice ages. Nature.

[15] Mangerud J (2004). Ice-dammed lakes and rerouting of the drainage of northern Eurasia during the Last Glaciation. Quat. Sci. Rev..

[16] DeWeese NE (2021). Early presence of *Bythotrephes cederströmii* (Cladocera: Cercopagidae) in lake sediments in North America: Evidence or artifact?. J. Paleolimnol..

[17] Litvinchuk LF, Rivier IK (2005). On history of systematics and distribution of representatives of the genus *Bythotrephes* (Polyphemoidea, Cladocera) on the territory of Russia and adjacent countries. Biologicheskie Resursy Vnutrennikh Vod: Bespozvonochnye.

[18] Litvinchuk LF, Litvinchuk SN (2016). Morphological diversity and widespread hybridization in the genus *Bythotrephes* Leydig, 1860 (Branchiopoda, Onychopoda, Cercopagidae). Arch. Biol. Sci..

[19] Kotov, A., Forró, L., Korovchinsky, N. M. & Petrusek, A. World checklist of freshwater Cladocera species. http://fada.biodiversity.be/group/show/17 (2013).

[20] Weider LJ (1991). Allozymic variation in *Bythotrephes cederstroemi*; A recent invader of the Great Lakes. J. Gt. Lakes Res..

[21] Berg DJ, Garton DW (1994). Genetic differentiation in North American and European populations of the cladoceran *Bythotrephes*. Limnol. Oceanogr..

[22] Berg DJ, Garton DW, MacIsaac HJ, Panov VE, Telesh IV (2002). Changes in genetic structure of North American *Bythotrephes* populations following invasion from Lake Ladoga, Russia. Freshw. Biol..

[23] Korovchinsky NM (2018). Further revision of the genus *Bythotrephes* Leydig (Crustacea: Cladocera: Onychopoda): Redescription of *B. brevimanus* Lilljeborg, reevaluation of *B. cederströmii* Schödler, and description of a new species of the genus. Zootaxa.

[24] deWaard JR (2006). Probing the relationships of the branchiopod crustaceans. Mol. Phylogenet. Evol..

[25] Flössner D (2000). Die Haplopoda und Cladocera (ohne Bosminidae) Mitteleuropas.

[26] Błędzki LA, Rybak JI (2016). Freshwater Crustacean Zooplankton of Europe.

[27] Korovchinsky NM (2019). Morphological assessment of the North Eurasian interspecific hybrid forms of the genus *Bythotrephes* Leydig, 1860 (Crustacea: Cladocera: Cercopagididae). Zootaxa.

[28] Mordukhai-Boltovskoi, F. D. & Rivier, I. K. Predatory cladocerans Podonidae, Polyphemidae, Cercopagidae, and Leptodoridae of the world fauna. In *Guide-Books on the Fauna of the USSR*, Vol. 148, 1–182 (Nauka, 1987).

[29] Grigorovich IA, Pashkova OV, Gromova YF, van Overdijk CDA (1998). *Bythotrephes longimanus* in the commonwealth of independent states: Variability, distribution and ecology. Hydrobiologia.

[30] Rivier IK, Grigorovich IA (1999). Biology of *Bythotrephes* Leydig (Crustacea, Cladocera, Onychopoda): Summary of research. Gidrobiologicheskii Zhurnal.

[31] Costa FO (2007). Biological identifications through DNA barcodes: The case of the Crustacea. Can. J. Fish. Aquat. Sci..

[32] Kotov AA, Taylor DJ (2010). A new African lineage of the *Daphnia obtusa* group (Cladocera: Daphniidae) disrupts continental vicariance patterns. J. Plankton Res..

[33] Janson K (1987). Allozyme and shell variation in two marine snails (*Littorina*, *Prosobranchia*) with different dispersal abilities. Biol. J. Linn. Soc..

[34] Trexler JC (1988). Hierarchical organization of genetic variation in the sailfin molly, *Poecilia lutipinna* (Pisces: Poeciliidae). Evolution.

[35] Lagercrantz U, Ryman N (1990). Genetic structure of Norway spruce (*Picea abies*): Concordance of morphological and allozymic variation. Evolution.

[36] Johannsson OE, Mills EL, O’Gorman R (1991). Changes in the nearshore and offshore zooplankton communities in Lake Ontario: 1981–88. Can. J. Fish. Aquat. Sci..

[37] Sprules WG, Riessen HP, Jin EH (1990). Dynamics of the *Bythotrephes* invasion of the St. Lawrence Great Lakes. J. Gt. Lakes Res..

[38] Colautti RI, Carlton GM, Ruiz JT (2003). Spatial and temporal analysis of transoceanic shipping vectors to the Great Lakes. Invasive Species: Vectors and Management Strategies.

[39] Kalendar R, Lee D, Schulman AH (2009). FastPCR software for PCR primer and probe design and repeat search. G3: Genes, Genom. Genom..

[40] Hall TA (1999). BioEdit: A user-friendly biological sequence alignment editor and analysis program for Windows 95/98/NT. Nucl. Acids Symp..

[41] Librado P, Rozas J (2009). DnaSP v5: A software for comprehensive analysis of DNA polymorphism data. Bioinformatics.

[42] Excoffier L, Lischer HEL (2010). Arlequin suite ver 3.5: A new series of programs to perform population genetics analyses under Linux and Windows. Mol. Ecol. Resour..

[43] Peakall R, Smouse PE (2006). GENALEX 6: Genetic analysis in excel. Population genetic software for teaching and research. Mol. Ecol. Notes.

[44] Tamura K, Stecher G, Peterson D, Filipski A, Kumar S (2013). MEGA6: Molecular evolutionary genetics analysis version 6.0. Mol. Biol. Evol..

[45] Hasegawa M, Kishino H, Yano T (1985). Dating of the human–ape splitting by a molecular clock of mitochondrial DNA. J. Mol. Evol..

[46] Akaike H (1973). Maximum likelihood identification of Gaussian autoregressive moving average models. Biometrika.

[47] Posada D (2008). jModelTest: Phylogenetic model averaging. Mol. Biol. Evol..

[48] Bandelt HJ, Forster P, Rohl A (1999). Median-joining networks for inferring intraspecific phylogenies. Mol. Biol. Evol..

