# Supplementary material for: New insight into the taxonomic resolution of the genus Bythotrephes Leydig (Crustacea: Cladocera) based on molecular data from Central Europe
Source: Sci Rep. 2021 Nov 30;11:23158. doi: 10.1038/s41598-021-02648-7 (PMC8633322; doi:10.1038/s41598-021-02648-7)
Supplement: Supplementary file 1 — Supplementary Information 1. [file 41598_2021_2648_MOESM1_ESM.docx]

**Supplementary Informations**

for the article ‘New insight into the taxonomic resolution of the genus *Bythotrephes* Leydig (Crustacea: Cladocera) based on molecular data from Central Europe’ by Maciej Karpowicz, Magdalena Świsłocka, Joanna Moroz, Łukasz Sługocki.

**Table S1.** Frequency of 30 mitochondrial DNA cytochrome c oxidase subunit 1 (COI) haplotypes (535 bp) found in the *Bythotrephes* lakes studied in Poland and Lithuania.

| **Haplotype Population** | **1** | **2** | **3** | **4** | **5** | **6** | **7** | **8** | **9** | **10** | **11** | **12** | **13** | **14** | **15** | **16** | **17** | **18** | **19** | **20** | **21** | **22** | **23** | **24** | **25** | **26** | **27** | **28** | **29** | **30** |
| --- | --- | --- | --- | --- | --- | --- | --- | --- | --- | --- | --- | --- | --- | --- | --- | --- | --- | --- | --- | --- | --- | --- | --- | --- | --- | --- | --- | --- | --- | --- |
| **Słowa** | **0** | **0** | **0** | **0** | **0** | **0** | **40.0** | **0** | **0** | **0** | **0** | **0** | **0** | **0** | **0** | **0** | **0** | **0** | **0** | **0** | **0** | **0** | **0** | **40.0** | **0** | **0** | **0** | **0** | **0** | **20.0** |
| **Ińsko** | **0** | **0** | **0** | **0** | **0** | **0** | **0** | **71.0** | **29.0** | **0** | **0** | **0** | **0** | **0** | **0** | **0** | **0** | **0** | **0** | **0** | **0** | **0** | **0** | **0** | **0** | **0** | **0** | **0** | **0** | **0** |
| **Lubie** | **25.0** | **25.0** | **0** | **0** | **0** | **0** | **0** | **0** | **0** | **0** | **25.0** | **0** | **0** | **0** | **0** | **0** | **0** | **0** | **0** | **0** | **0** | **0** | **0** | **25.0** | **0** | **0** | **0** | **0** | **0** | **0** |
| **Krzemno** | **0** | **0** | **0** | **0** | **0** | **0** | **0** | **0** | **0** | **0** | **0** | **0** | **0** | **0** | **0** | **0** | **0** | **0** | **0** | **0** | **0** | **0** | **0** | **0** | **80.0** | **20.0** | **0** | **0** | **0** | **0** |
| **Drawsko** | **0** | **0** | **0** | **0** | **37.5** | **12.5** | **0** | **0** | **0** | **0** | **0** | **0** | **0** | **0** | **0** | **0** | **0** | **0** | **0** | **0** | **0** | **0** | **50.0** | **0** | **0** | **0** | **0** | **0** | **0** | **0** |
| **Żerdno** | **0** | **0** | **20.0** | **0** | **20.0** | **0** | **20.0** | **0** | **0** | **0** | **0** | **0** | **0** | **0** | **0** | **0** | **0** | **0** | **0** | **0** | **0** | **0** | **40.0** | **0** | **0** | **0** | **0** | **0** | **0** | **0** |
| **Pile** | **0** | **0** | **0** | **0** | **0** | **0** | **0** | **0** | **0** | **0** | **0** | **0** | **0** | **0** | **0** | **0** | **0** | **0** | **0** | **0** | **0** | **0** | **0** | **0** | **0** | **0** | **0** | **0** | **100** | **0** |
| **Trzesiecko** | **0** | **0** | **0** | **0** | **0** | **0** | **0** | **0** | **0** | **0** | **0** | **0** | **0** | **0** | **0** | **0** | **0** | **0** | **0** | **0** | **0** | **0** | **0** | **0** | **0** | **0** | **33.3** | **66.7** | **0** | **0** |
| **Szelment Wielki** | **0** | **0** | **0** | **0** | **0** | **0** | **0** | **0** | **0** | **0** | **83.3** | **0** | **0** | **0** | **0** | **0** | **16.7** | **0** | **0** | **0** | **0** | **0** | **0** | **0** | **0** | **0** | **0** | **0** | **0** | **0** |
| **Garbaś** | **60.0** | **0** | **0** | **0** | **0** | **0** | **0** | **0** | **0** | **0** | **0** | **0** | **0** | **0** | **0** | **0** | **0** | **40.0** | **0** | **0** | **0** | **0** | **0** | **0** | **0** | **0** | **0** | **0** | **0** | **0** |
| **Hańcza** | **60.0** | **0** | **20.0** | **0** | **0** | **0** | **0** | **0** | **0** | **0** | **0** | **0** | **0** | **0** | **10.0** | **10.0** | **0** | **0** | **0** | **0** | **0** | **0** | **0** | **0** | **0** | **0** | **0** | **0** | **0** | **0** |
| **Białe Wigierskie** | **0** | **0** | **0** | **0** | **0** | **0** | **83.3** | **0** | **0** | **16.7** | **0** | **0** | **0** | **0** | **0** | **0** | **0** | **0** | **0** | **0** | **0** | **0** | **0** | **0** | **0** | **0** | **0** | **0** | **0** | **0** |
| **Busznica** | **0** | **16.7** | **0** | **0** | **0** | **0** | **0** | **0** | **0** | **0** | **0** | **0** | **0** | **0** | **0** | **0** | **0** | **0** | **0** | **0** | **83.3** | **0** | **0** | **0** | **0** | **0** | **0** | **0** | **0** | **0** |
| **Wigry** | **77.8** | **0** | **0** | **0** | **0** | **0** | **0** | **0** | **0** | **0** | **0** | **0** | **0** | **0** | **0** | **0** | **0** | **0** | **11.1** | **11.1** | **0** | **0** | **0** | **0** | **0** | **0** | **0** | **0** | **0** | **0** |
| **Studzienniczne** | **0** | **20.0** | **60.0** | **20.0** | **0** | **0** | **0** | **0** | **0** | **0** | **0** | **0** | **0** | **0** | **0** | **0** | **0** | **0** | **0** | **0** | **0** | **0** | **0** | **0** | **0** | **0** | **0** | **0** | **0** | **0** |
| **Serwy** | **0** | **0** | **100** | **0** | **0** | **0** | **0** | **0** | **0** | **0** | **0** | **0** | **0** | **0** | **0** | **0** | **0** | **0** | **0** | **0** | **0** | **0** | **0** | **0** | **0** | **0** | **0** | **0** | **0** | **0** |
| **Gaładuś** | **0** | **0** | **71.4** | **0** | **0** | **0** | **0** | **14.3** | **0** | **0** | **0** | **14.3** | **0** | **0** | **0** | **0** | **0** | **0** | **0** | **0** | **0** | **0** | **0** | **0** | **0** | **0** | **0** | **0** | **0** | **0** |
| **Galstas** | **0** | **0** | **0** | **0** | **0** | **0** | **0** | **0** | **0** | **0** | **0** | **0** | **0** | **100** | **0** | **0** | **0** | **0** | **0** | **0** | **0** | **0** | **0** | **0** | **0** | **0** | **0** | **0** | **0** | **0** |
| **Ancia** | **0** | **0** | **0** | **0** | **0** | **0** | **0** | **0** | **0** | **0** | **20.0** | **0** | **0** | **0** | **0** | **0** | **0** | **0** | **0** | **0** | **0** | **80.0** | **0** | **0** | **0** | **0** | **0** | **0** | **0** | **0** |
| **Aviris** | **0** | **0** | **0** | **0** | **0** | **0** | **0** | **0** | **0** | **0** | **0** | **0** | **100** | **0** | **0** | **0** | **0** | **0** | **0** | **0** | **0** | **0** | **0** | **0** | **0** | **0** | **0** | **0** | **0** | **0** |

**Table S2.** Values of genetic differentiation of mitochondrial DNA cytochrome c oxidase subunit 1 (COI) gene (535 bp) of *Bythotrephes* between lake pairs in Poland (17 lakes) and Lithuania (3 lakes), as measured by *Φ*_ST_ (above the diagonal) and *F*_ST_ (below the diagonal).

| Lake | 1 | 2 | 3 | 4 | 5 | 6 | 7* | 8 | 9 | 10 | 11 | 12 | 13 | 14 | 15 | 16 | 17 | 18 | 19 | 20 |
| --- | --- | --- | --- | --- | --- | --- | --- | --- | --- | --- | --- | --- | --- | --- | --- | --- | --- | --- | --- | --- |
| 1 | – | 0.711 | 0.247 | 0.533 | 0.832 | 0.419 | 0.733 | 0.396 | 0.820 | 0.802 | 0.681 | 0.200 | 0.883 | 0.751 | 0.891 | 0.904 | 0.632 | 0.835 | 0.711 | 0.928 |
| 2 | 0.381 | – | 0.326 | 0.509 | 0.831 | 0.358 | 0.935 | 0.556 | 0.833 | 0.871 | 0.701 | 0.833 | 0.912 | 0.794 | 0.927 | 0.957 | 0.600 | 0.815 | 0.716 | 0.972 |
| 3 | 0.006 | 0.311 | – | 0.282 | 0.572 | 0.066 | 0.128 | 0.103 | 0.404 | 0.217 | 0.159 | 0.357 | 0.449 | 0.246 | 0.399 | 0.335 | 0.112 | 0.414 | 0.167 | 0.580 |
| 4 | 0.400 | 0.557 | 0.326 | – | 0.721 | 0.365 | 0.474 | 0.392 | 0.663 | 0.702 | 0.637 | 0.610 | 0.790 | 0.696 | 0.788 | 0.773 | 0.546 | 0.646 | 0.599 | 0.827 |
| 5 | 0.269 | 0.418 | 0.191 | 0.438 | – | 0.122 | 0.925 | 0.713 | 0.895 | 0.895 | 0.768 | 0.895 | 0.906 | 0.848 | 0.917 | 0.936 | 0.682 | 0.906 | 0.804 | 0.964 |
| 6 | 0.076 | 0.335 | 0.053 | 0.350 | 0.000 | – | 0.429 | 0.099 | 0.552 | 0.598 | 0.497 | 0.483 | 0.619 | 0.603 | 0.583 | 0.539 | 0.273 | 0.515 | 0.452 | 0.761 |
| 7* | 0.200 | 0.524 | 0.000 | 0.600 | 0.321 | 1.000 | – | 0.636 | 0.964 | 0.830 | 0.664 | 0.936 | 0.922 | 0.776 | 0.938 | 1.000 | 0.596 | 1.000 | 0.684 | 1.000 |
| 8 | 0.253 | 0.462 | 0.147 | 0.499 | 0.325 | 0.195 | 0.333 | – | 0.763 | 0.773 | 0.620 | 0.550 | 0.829 | 0.726 | 0.839 | 0.852 | 0.467 | 0.774 | 0.609 | 0.926 |
| 9 | 0.450 | 0.591 | 0.211 | 0.636 | 0.476 | 0.402 | 0.667 | 0.554 | – | 0.899 | 0.743 | 0.923 | 0.933 | 0.833 | 0.947 | 0.977 | 0.681 | 0.869 | 0.754 | 0.986 |
| 10 | 0.300 | 0.470 | 0.074 | 0.500 | 0.355 | 0.250 | 0.400 | 0.374 | 0.544 | – | 0.041 | 0.872 | 0.749 | 0.000 | 0.758 | 0.791 | 0.482 | 0.915 | 0.518 | 0.823 |
| 11 | 0.292 | 0.430 | 0.072 | 0.449 | 0.340 | 0.218 | 0.356 | 0.349 | 0.484 | 0.024 | – | 0.716 | 0.482 | 0.072 | 0.362 | 0.366 | 0.269 | 0.745 | 0.374 | 0.600 |
| 12 | 0.167 | 0.591 | 0.385 | 0.636 | 0.476 | 0.280 | 0.667 | 0.554 | 0.667 | 0.544 | 0.484 | – | 0.933 | 0.795 | 0.947 | 0.977 | 0.681 | 0.952 | 0.778 | 0.982 |
| 13 | 0.450 | 0.591 | 0.356 | 0.636 | 0.476 | 0.402 | 0.667 | 0.554 | 0.667 | 0.544 | 0.484 | 0.667 | – | 0.705 | 0.740 | 0.803 | 0.432 | 0.948 | 0.624 | 0.927 |
| 14 | 0.430 | 0.556 | 0.197 | 0.590 | 0.457 | 0.387 | 0.583 | 0.512 | 0.618 | 0.066 | 0.003 | 0.618 | 0.618 | – | 0.700 | 0.717 | 0.509 | 0.837 | 0.532 | 0.745 |
| 15 | 0.250 | 0.426 | 0.116 | 0.450 | 0.312 | 0.091 | 0.300 | 0.313 | 0.497 | 0.350 | 0.242 | 0.497 | 0.479 | 0.471 | – | 0.000 | 0.044 | 0.964 | 0.633 | 0.949 |
| 16 | 0.502 | 0.666 | 0.429 | 0.743 | 0.524 | 0.318 | 1.000 | 0.667 | 0.771 | 0.620 | 0.428 | 0.771 | 0.771 | 0.690 | 0.000 | – | 0.000 | 1.000 | 0.620 | 1.000 |
| 17 | 0.354 | 0.443 | 0.281 | 0.530 | 0.396 | 0.189 | 0.476 | 0.429 | 0.566 | 0.443 | 0.312 | 0.566 | 0.566 | 0.535 | 0.000 | 0.000 | – | 0.673 | 0.348 | 0.688 |
| 18 | 0.661 | 0.762 | 0.627 | 0.836 | 0.642 | 0.616 | 1.000 | 0.816 | 0.847 | 0.750 | 0.632 | 0.847 | 0.847 | 0.767 | 0.706 | 1.000 | 0.738 | – | 0.773 | 1.000 |
| 19 | 0.400 | 0.557 | 0.289 | 0.600 | 0.438 | 0.350 | 0.600 | 0.500 | 0.563 | 0.500 | 0.449 | 0.636 | 0.636 | 0.590 | 0.450 | 0.743 | 0.530 | 0.835 | – | 0.766 |
| 20 | 0.634 | 0.744 | 0.593 | 0.819 | 0.620 | 0.586 | 1.000 | 0.793 | 0.833 | 0.727 | 0.613 | 0.833 | 0.833 | 0.752 | 0.681 | 1.000 | 0.719 | 1.000 | 0.820 | – |

Significant values are underlined; * – due to only one individual, Pile Lake was not included in statistical comparisons.


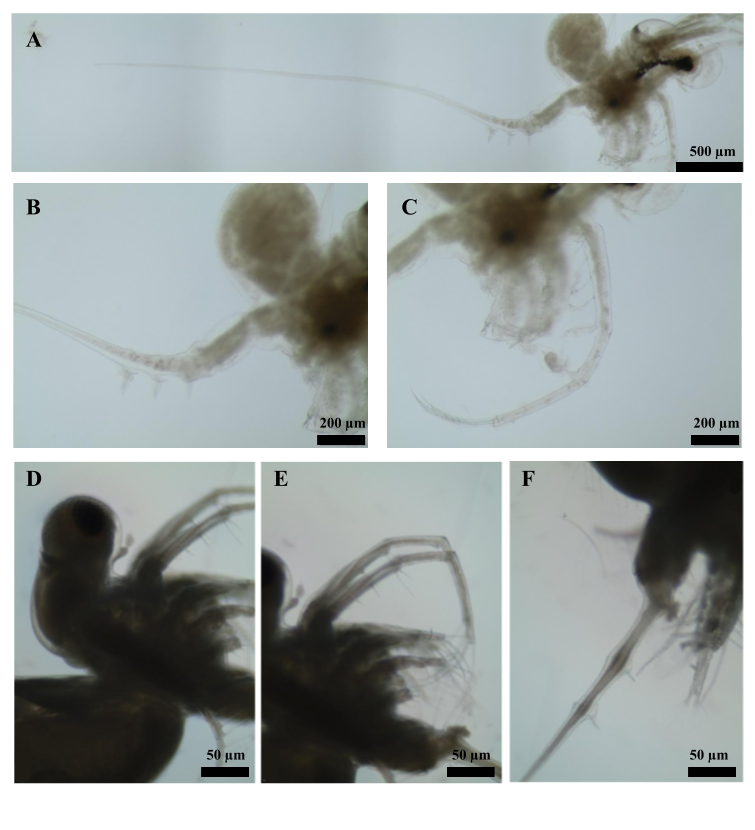


**Figure S1**. Difference in morphological traits for two populations with the same haplotype (H7) from Lake Białe Wigierskie (A, B, C, BOLD sample ID: UwB_17_Bl17), and Lake Słowa (D, E, F, BOLD sample ID: UwB_112_Bl112).
